# Effects of SRW^®^ Stem on the functional properties, oxidative stress and inflammation of human umbilical cord mesenchymal stem cells

**DOI:** 10.29219/fnr.v70.14068

**Published:** 2026-05-25

**Authors:** Yoann Birling, Greg Macpherson, Jonathan Lim, Hongbin Qi, Deep Bhuyan, Julie Chen

**Affiliations:** 1 Wellizen Australia, Sydney, Australia; 2 Science Research Wellness Laboratories, Auckland, New Zealand; 3 Shanghai Huabo Biotechnology Co., Ltd, Shanghai, China; 4 NICM Health Research Institute, Western Sydney University, Penrith, Australia

**Keywords:** stem cell, ageing, anti-ageing, mitochondria, oxidative stress, inflammation, mitoquinol, proliferation, migration, differentiation

## Abstract

**Background:**

Global population ageing has heightened the demand for anti-ageing nutritional interventions. Science Research Wellness (SRW^®^) Stem was designed by SRW^®^ to address this demand with ingredients that improve stem cell function, which is central to protection against age-related decline. These ingredients include mitoquinol, sea buckthorn extract, fucoidan, oleuropein, and vitamin D3.

**Objectives:**

We compared the effects of SRW^®^ Stem on the functions of human umbilical cord mesenchymal stem cells (hUC-MSCs) with those of mitoquinol alone, which is known to improve these functions.

**Design:**

*In vitro* assays, including CCK-8 for cell viability, trypan blue exclusion, colony formation, transwell migration, adipogenic differentiation, ROS/glutathione analysis, and cytokine profiling under oxidative stress, were used to assess proliferation, migration, and differentiation capacities, as well as antioxidant and anti-inflammatory activity of the SRW^®^ Stem.

**Results:**

Results demonstrated that SRW^®^ Stem 5 and 10 μg/mL significantly (*P* < 0.05 for all) enhanced proliferation, colony formation, and migration, and promoted adipogenic differentiation in the hUC-MSCs, compared with vehicle control and mitoquinol (positive control). Under oxidative stress, SRW^®^ Stem significantly reduced (*P* < 0.001 for all) intracellular ROS, increased glutathione synthesis, suppressed IL-1β secretion, and restored IL-10 production in hUC-MSCs.

**Discussions:**

These findings indicated that SRW^®^ Stem 5 and 10 μg/mL exerts antioxidant, anti-inflammatory, and pro-regenerative effects on hUC-MSCs, outperforming mitoquinol across most parameters.

**Conclusions:**

The results highlighted the potential of SRW^®^ Stem as an anti-ageing intervention and a supportive agent in stem cell-based therapies. Further investigations in animal models and clinical trials are warranted to validate its efficacy.

## Popular scientific summary

- As the global population ages, researchers are exploring ways to support healthy ageing by improving stem cell function. Stem cells are specialised cells that can self-renew and develop into different cell types, playing a key role in tissue repair and regeneration, but their function declines with age due to oxidative stress and inflammation. In this laboratory study, scientists tested SRW^®^ Stem, a nutritional supplement containing natural ingredients such as mitoquinol, sea buckthorn extract, fucoidan, oleuropein, and vitamin D3, on human stem cells. Compared with untreated cells and mitoquinol alone, the supplement improved stem cell growth, migration, and regenerative capacity, while also reducing oxidative stress and inflammation. These findings suggest that SRW^®^ Stem may help protect stem cells from age-related decline and could have potential as a future anti-ageing or regenerative health intervention, although further animal and human studies are needed.

Global demographics are undergoing a profound transformation as people around the world live longer than ever before (1). According to the World Health Organization (1), the number of people aged 60 years or older will reach 1.4 billion by 2030 – representing one in six individuals globally – and is projected to double to 2.1 billion by 2050. The number of those aged 80 years or older is expected to triple to 426 million (2). Today, the prospect of reaching the age of 60 – and well beyond – has become a common expectation for most individuals. Consequently, this increase in life expectancy also accentuates the problems and health conditions associated with ageing. Several conditions associated with ageing, notably cardiovascular diseases, diabetes, frailty, osteoarthritis and cancer have seen a rising prevalence in recent years (3–5).

There is an increasing desire worldwide not only to live longer, but also to live healthier and longer (6). Ageing is the result of the gradual accumulation of diverse molecular and cellular damage over time (2), leading to a progressive decline in physiological and cognitive capacities, a mounting vulnerability to diseases and, ultimately, mortality (7). Since the deterioration of ageing health is largely attributed to molecular and cellular damage over time, the basic strategy for anti-ageing targets restoring damaged cells and removing senescent cells. Stem cells play a key role in cell rejuvenation and the prevention of age-related biological deterioration (8).

Stem cells are undifferentiated cells that possess the unique capability to self-renew and differentiate into specialised cell types, making them the biological foundation for tissue regeneration, repair, and maintenance of metabolic homeostasis (9, 10). Stem cells are responsible for replenishing aged or damaged cells in many tissues, helping to maintain organ function and delay the effects of ageing (11). As people age, stem cells progressively lose their proliferative and regenerative capacity due to the accumulation of DNA damage, telomere shortening, epigenetic drift, and mitochondrial impairment (12). This reduction in regenerative potential is a core factor in age-related diseases and diminished organ function (13–17).

Nutrition and targeted supplementation profoundly impact stem cell function and overall regenerative capacity. Supplements such as omega-3 fatty acids, which are known for their anti-inflammatory effects (18–20), vitamin D3, which has antioxidant effects (21, 22), resveratrol, which enhances DNA repair (23–26) and curcumin, which protects cells against inflammation and oxidative stress (27–30), are recognised for their capacity to support stem cell health and may aid in creating an optimal environment for cellular regeneration and recovery (31). NAD^+^ Precursors (NR/NMN), which enhance mitochondrial metabolism and stem cell vitality by restoring NAD^+^ levels and activating sirtuins have also been investigated (32, 33).

Science Research Wellness (SRW^®^), a biotechnology company based in New Zealand, has developed a wellness product called Stem to improve stem cell function. These ingredients (see Supplementary Material 1 for the full composition) are mitoquinol mesylate, sea buckthorn extract, fucoidan, Oleuropein, and Vitamin D3. Mitoquinol is a synthetic analogue of ubiquinone with strong antioxidant effects targeting the mitochondria (34). Mitoquinol has been widely researched for its ability to improve cell viability and function, including stem cell function (35) and has beneficial effects on various aspects of health in humans (36–39), which is why it was selected as a positive control in this study. The other ingredients in the product, such as sea buckthorn extract, which is rich in proanthocyanidin, Fucoidan, a sulphated polysaccharide mainly extracted from brown marine algae, Oleuropein, a secoiridoid glycoside extracted from olive leaves and Vitamin D3, have antioxidant, anti-ageing, anti-inflammatory effects and promote growth, mitochondrial health and DNA protection in various cells (40–43) and may also have beneficial effects on stem cell proliferation and differentiation (44–48).

Taken together, these studies suggest that SRW^®^ Stem may improve the function of stem cells, with potential applications in anti-ageing and recovery from injury and surgery. However, the effect of SRW^®^ Stem on various aspects of stem cell health has not been evaluated. In particular, we do not know the effect of SRW^®^ Stem on stem cell differentiation, which is impaired by senescence in the ageing process (49). Additionally, whether the effects of the combination are superior to mitoquinol alone is unknown. The objectives of this study were to assess the effect of SRW^®^ Stem on stem cell health, including proliferation, migration, differentiation, and anti-inflammatory and antioxidant functions, to determine whether SRW^®^ Stem may support the body’s own stem cell function and health to promote cellular rejuvenation and healthy ageing. Therefore, the ultimate objective of this study is to assess the potential of SRW^®^ Stem as an anti-ageing product and to support and guide further research into this potential effect.

## Methods

### Cell culture

Primary human umbilical cord mesenchymal stem cells (hUC-MSCs, ATCC PCS-500-010, sourced from Gactoo company, Shanghai, China) were used in this study, as their multipotency exemplifies the broader potential of stem cells. The cells were cultured in a serum-free complete medium specifically designed for hUC-MSCs (Gactoo, Shanghai, China) using methods described elsewhere (49).

### Test substances preparation

The working concentrations of mitoquinol mesylate and SRW^®^ Stem were determined using a CCK-8 cell viability assay, following the methods of a previous study (50). A total of 40 mg of mitoquinol mesylate (Rhawn Company, Shanghai, China) and SRW^®^ Stem were weighed and dissolved in 1 mL of dimethyl sulfoxide (DMSO) each to prepare stock solutions at a concentration of 40 mg/mL. The stock solutions were diluted to the required concentrations with complete culture medium before use. The test substances were stored at 4°C and used directly after preparation to avoid denaturation during storage at −20°C due to the high DMSO content.

### Assessment of hUC-MSC viability by CCK-8 assay

Healthy hUC-MSCs were seeded into 96-well plates at a density of 5 × 10^3^ cells per well. When the cell confluency reached approximately 70%, the medium was replaced with complete medium containing mitoquinol mesylate and SRW^®^ Stem at concentrations of 0.5, 1, 5, 10, 20, 50, 100, 200, 400, 800 and 1,000 μg/mL. The cells were incubated for an additional 24 h (37°C, 5% CO_2_, and 95% humidity). After washing with phosphate-buffered saline (PBS), complete medium containing 10% CCK-8 reagent (APExBio, Houston, Texas, USA) was added, and the cells were incubated at 37°C for 2 h. The optical density (OD) of each well was then measured at 450 nm using a microplate reader (Thermo Fisher, Waltham, Massachusetts, USA). This test was used as a basis for selecting the ideal concentrations in subsequent tests.

### Assessment of hUC-MSC viability by Trypan Blue Exclusion Assay

The Trypan Blue Exclusion Assay was used to confirm the effect of SRW^®^ Stem on cell viability after selecting the optimal concentration with the CCK-8 Assay (51). Healthy hUC-MSCs were seeded into 12-well plates. When the cell confluency reached approximately 50%, the medium was replaced with complete medium containing different concentrations of mitoquinol mesylate (5 and 1 μg/mL) and SRW^®^ Stem (10 and 5 μg/mL), as determined by the CCK-8 Assay. Cells were incubated for an additional 24 h (37°C, 5% CO_2_, and 95% humidity). After washing with PBS, cells were harvested by trypsinisation and collected by centrifugation. The supernatant was discarded, and the cell pellet was resuspended and stained with Trypan Blue solution (Trypan Blue: complete medium = 1:1) for 3 min. The percentage of viable cells was determined by counting the number of viable cells and the total number of cells per field under a microscope (EVOS M7000, Thermo Fisher, Waltham, Massachusetts, USA).

### Colony-Forming Unit Assay

Exponentially growing hUC-MSCs were harvested by routine trypsinisation and collected by centrifugation. The cell pellet was resuspended and counted, and the cell density was adjusted to 5 × 10^3^ cells/mL. Culture medium containing mitoquinol mesylate (5 and 1 μg/mL) and SRW^®^ Stem (10 and 5 μg/mL) was added to separate wells of a 6-well plate. A 100 μL cell suspension (approximately 500 cells) was seeded into each well and cultured under standard conditions until visible cell colonies formed. Once visible colonies were observed, the culture was terminated, and the medium was discarded. Wells were gently washed three times with pre-chilled PBS (4°C). Each well was then fixed with 2 mL of 4% paraformaldehyde for 10 min, followed by three washes in PBS. Subsequently, 2 mL of 0.1% crystal violet solution was added to each well for 5 min to stain the colonies (52). The dye was gently rinsed off under running water, and excess water was removed from the 6-well plate before photographing and counting the colonies.

### Cell migration assay

The hUC-MSCs in the logarithmic growth phase were digested with trypsin and resuspended in serum-free medium (53). The cell density was adjusted to 2 × 10^5^ cells/mL. Transwell inserts were placed into a 24-well plate. The lower chamber was filled with culture medium containing mitoquinol mesylate (5 and 1 μg/mL) and SRW^®^ Stem (10 and 5 μg/mL), while 100 μL of serum-free medium was added to the upper chamber. Next, 50 μL of the cell suspension (approximately 10,000 cells) was seeded into the upper chamber of each Transwell insert. After incubation under standard culture conditions for 36 h, the migrated cells were fixed with paraformaldehyde and stained with crystal violet. The number of migrated cells was then counted under a microscope (Thermo Fisher, Waltham, Massachusetts, USA).

### Adipogenic differentiation of hUC-MSCs

The hUC-MSCs in the logarithmic growth phase were passed into 6-well plates. When the cell confluence reached approximately 90%, the medium was replaced with adipogenic differentiation induction medium (HUXUC-90031, Cyagen Biosciences, Suzhou, Jiangsu, China) containing mitoquinol mesylate (5 and 1 μg/mL) and SRW^®^ Stem (10 and 5 μg/mL). According to the manufacturer’s instructions for the adipogenic induction medium, cells were treated with Solution A (which contains the basal medium, foetal bovine serum, and differentiation supplements A-I and A-II) for 3 days, then switched to Solution B (which contains the basal medium, foetal bovine serum, and the differentiation supplement B) for 1 day, and this cycle was repeated until the formation of lipid droplets was observed.

At the end of differentiation, the cells were gently washed with PBS, fixed with Oil Red O fixative solution at room temperature for 30 min, and then the fixative was discarded. Cells were washed twice with distilled water, stained with freshly prepared Oil Red O staining solution for 20 min, and washed three times with distilled water until no residual dye remained. Finally, the cells were covered with distilled water, observed, and photographed under a microscope (Thermo Fisher, Waltham, Massachusetts, USA) to document lipid droplet formation.

### Measurement of intracellular reactive oxygen species, glutathione, and inflammatory cytokines under H_2_O_2_ stimulation

The hUC-MSCs in the logarithmic growth phase were seeded into 24-well plates and cultured under standard conditions until the cell density reached approximately 40%. Except for the negative control group (untreated) and the positive control group (treated with 300 μM H_2_O_2_ only), the culture medium was replaced with medium containing mitoquinol mesylate (5 and 1 μg/mL) and SRW^®^ Stem (10 and 5 μg/mL), followed by incubation for 24 h. Subsequently, 300 μM H_2_O_2_ was added to all groups except the negative control group, and cells were incubated for an additional 12 h (54).

For reactive oxygen species (ROS) detection, after incubation, intracellular ROS were measured using a ROS assay kit (Cat:s S0033S, Beyotime, Shanghai, China). The ROS fluorescent probe was loaded according to the kit instructions, incubated at 37 °C for 20 min, and washed three times with PBS. Cells were then observed and imaged under a fluorescence microscope, and the mean fluorescence intensity was quantified using ImageJ software (Thermo Fisher, Waltham, Massachusetts, USA).

For glutathione (GSH) measurement, cells were washed with PBS, collected, and intracellular total GSH was extracted using the Total Glutathione Assay Kit (Cat: S0052, Beyotime, Shanghai, China). The GSH content was measured according to the manufacturer’s instructions, and the absorbance was recorded at 412 nm using a microplate reader (Multiskan SkyHigh, Thermo Fisher, Waltham, Massachusetts, USA).

For interleukin-1β (IL-1β) and interleukin-10 (IL-10) detection, the culture supernatant was collected at the end of treatment. The levels of IL-1β and IL-10 in the medium were determined using specific ELISA kits (IL-10 Cat:CSB-E04593h, IL-1β Cat:CSB-E08053h, Huamei Bio, Wuhan, Hubei, China) according to the manufacturer’s protocols.

### Statistical analysis

Differences were analysed using one-way ANOVA (Dunnett’s test or Tukey post hoc test) for multiple comparisons, and comparisons between two groups were analysed using two-tailed unpaired Student’s t-test. Data are presented as mean ± SEM unless otherwise noted. The differences with a statistical significance level of *P* < 0.05 were considered statistically significant. All the tests were conducted with SPSS 31.0.1.0 (IBM Corporation, Armonk, New York, United States).

## Results

### Effects on cell viability

**Fig. 1:**
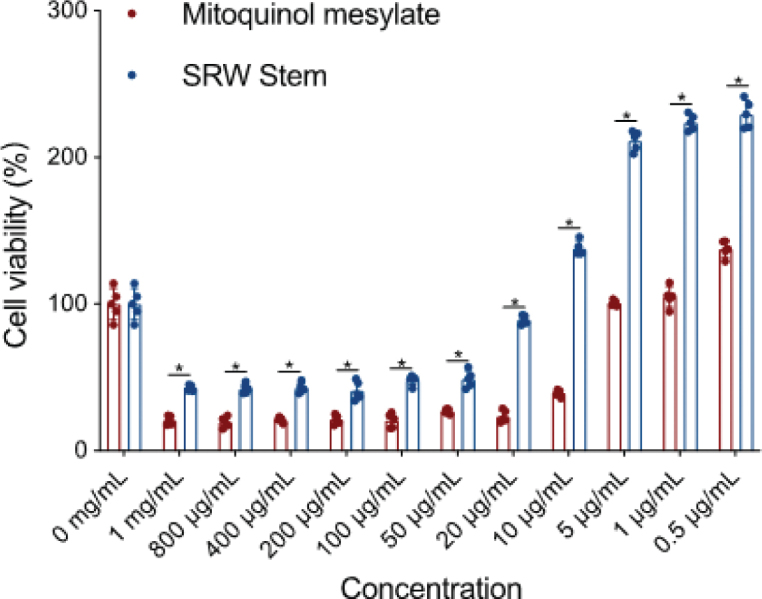
CCK-8 assay showing the effects of various concentrations of mitoquinol mesylate and SRW^®^ Stem on hUC-MSC viability compared with the untreated control. Mitoquinol mesylate is presented in red and SRW^®^ Stem in blue. The asterisk ‘*’ refers to a *P*-value of less than 0.05. *n* = 5.

To determine the optimal working concentrations of mitoquinol Mesylate and SRW^®^ Stem, a CCK-8 assay was performed to evaluate the viability of the hUC-MSCs after 24-h treatment with different concentrations of each compound. As shown in Fig. 1, compared to the untreated control, high concentrations of mitoquinol mesylate (1 mg/mL – 10 μg/mL) and SRW^®^ Stem (1 mg/mL – 20 μg/mL) significantly reduced cell viability (*P* < 0.05). Concentrations of 5 and 1 μg/mL mitoquinol mesylate showed no significant effect on cell viability (*P* > 0.05), remaining comparable to the control group, while 0.5 μg/mL mitoquinol mesylate slightly increased cell viability and promoted cell growth (*P* < 0.001). Similarly, SRW^®^ Stem at concentrations ranging from 10 to 0.5 μg/mL enhanced cell viability and promoted cell proliferation. Compared with mitoquinol mesylate, SRW^®^ Stem exhibited lower cytotoxicity (*P* < 0.05) based on cell viability relative to the untreated control across all concentrations tested (see Fig. 1). Based on these results, concentrations of 5 and 1 μg/mL mitoquinol mesylate, as well as 10 and 5 μg/mL SRW^®^ Stem, were selected for subsequent experiments with hUC-MSCs.

**Fig. 2:**
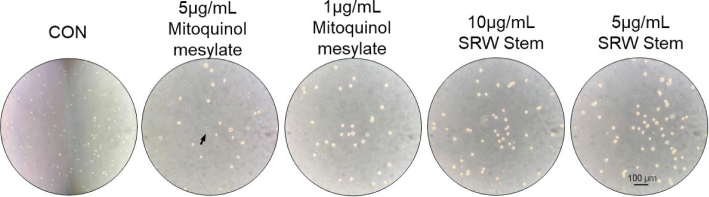
Representative trypan blue staining images of human umbilical cord mesenchymal stem cells treated with different concentrations of mitoquinol mesylate and SRW^®^ Stem. The arrows indicate dead cells, which are stained blue; *n* = 5.

To further determine the effects of mitoquinol mesylate and SRW^®^ Stem on cell viability, the hUC-MSCs were treated with mitoquinol mesylate at concentrations of 5 and 1 μg/mL, and SRW^®^ Stem at concentrations of 10 and 5 μg/mL for 24 h. As shown in Fig. 2, trypan blue exclusion assay revealed that treatment with 5 μg/mL mitoquinol mesylate resulted in a slight reduction in cell viability (viable cell percentage: 97.77%), whereas treatment with 1 μg/mL mitoquinol mesylate or 10 and 5 μg/mL SRW^®^ Stem did not affect cell viability (viable cell percentage: 100%).

### Effects on proliferative capacity

**Fig. 3:**
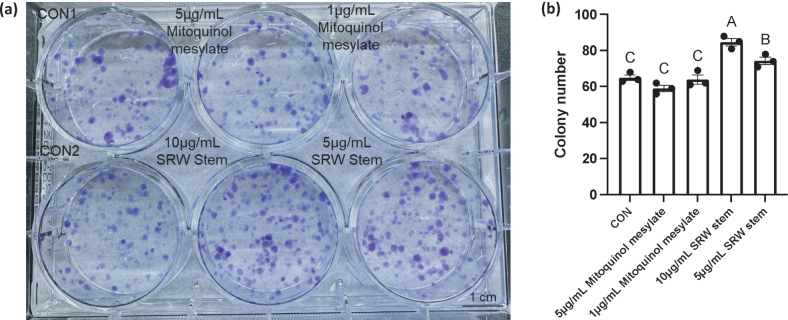
Colony formation of human umbilical cord mesenchymal stem cells under treatment with mitoquinol mesylate and SRW^®^ Stem. Image A shows the most representative image of the results and Image (a) shows the average of the three experiments, *n* = 3. The letters on the graph (b) represent the statistical significance of differences; results sharing the same letter have a difference of *P* > 0.05 and results with different letters have a difference of *P* < 0.05.

The effect of mitoquinol mesylate and SRW^®^ Stem on stem cell proliferation was determined with a Colony-Forming Unit (CFU) assay. As shown in Fig. 3, mitoquinol mesylate at concentrations of 5 and 1 μg/mL did not significantly affect the colony-forming ability of hUC-MSCs (*P* > 0.05), with colony numbers comparable to those in the untreated control group. In contrast, SRW^®^ Stem significantly promoted colony formation (*P* < 0.05 for both concentrations), with 10 μg/mL SRW^®^ Stem demonstrating a stronger promotive effect than 5 μg/mL SRW^®^ Stem.

### Effects on migratory capacity

**Fig. 4:**
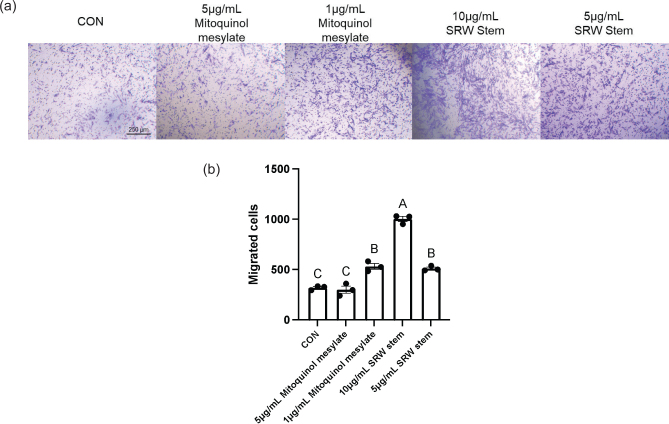
Effects of mitoquinol mesylate and SRW^®^ Stem on the Migration of Human Umbilical Cord Mesenchymal Stem Cells. Image (a) represents the microscopic photographs of the cell cultures and graph (b) represents the number of migrated cells. The letters on graph (b) represent the statistical significance of differences; results sharing the same letter have a difference of *P* > 0.05 and results with different letters have a difference of *P* < 0.05.

The effects of mitoquinol mesylate and SRW^®^ Stem on the migratory capacity of the hUC-MSCs were tested with a Transwell cell migration assay. As shown in Fig. 4, treatment with 5 μg/mL mitoquinol mesylate resulted in a slight decrease in the number of migrated cells compared with the untreated control group, but the difference was not significant. In contrast, treatment with 1 μg/mL mitoquinol mesylate, 10 μg/mL SRW^®^ Stem, and 5 μg/mL SRW^®^ Stem significantly increased the number of migrated cells compared with the control (*P* < 0.01), with 10 μg/mL SRW^®^ Stem demonstrating the most pronounced effect. The effects of 1 μg/mL mitoquinol mesylate and 5 μg/mL SRW^®^ Stem were statistically similar (*P* = 0.97). These results suggested that 1 μg/mL mitoquinol mesylate and both concentrations of SRW^®^ Stem (particularly 10 μg/mL) can promote the migration of hUC-MSCs compared to the untreated control.

### Effects on adipogenic differentiation

**Fig. 5:**
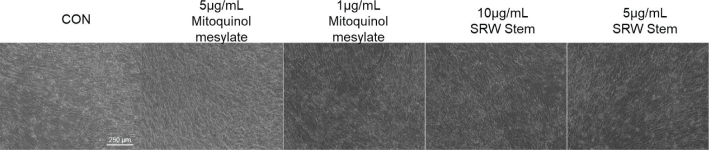
Effect of 5 and 1 μg/mL Mitoquinol Mesylate and 10 and 5 μg/mL SRW^®^ Stem on Human Umbilical Cord Mesenchymal Stem Cells after 28 Days of Adipogenic Induction (post-staining). CON, untreated control.

The effects of mitoquinol mesylate and SRW^®^ Stem on the adipogenic differentiation of hUC-MSCs that were treated with an adipogenic induction medium were assessed by visual observation. As shown in Fig. 5, only a small number of lipid droplets were observed in the control group and in the groups treated with 5 and 1 μg/mL mitoquinol mesylate. In contrast, based on the morphological examination, the groups treated with 10 and 5 μg/mL SRW^®^ Stem exhibited increased lipid droplet formation, with the 10 μg/mL SRW^®^ Stem group showing a more pronounced effect. These results indicated that mitoquinol mesylate had no apparent effect on adipogenic differentiation of hUC-MSCs, whereas SRW^®^ Stem promoted adipogenic differentiation at both tested concentrations.

### Effects on oxidative stress

To investigate the antioxidant effects of mitoquinol mesylate and SRW^®^ Stem, the intracellular ROS and GSH levels of hUC-MSCs under H_2_O_2_-induced oxidative stress were measured.

**Fig. 6:**
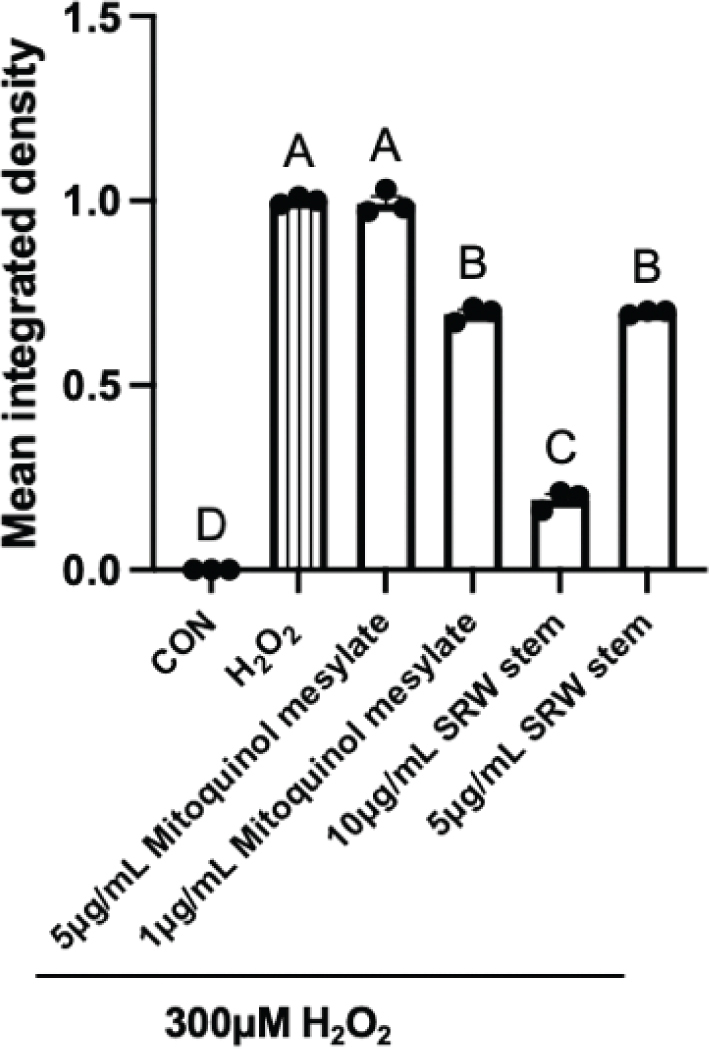
Effects of mitoquinol mesylate and SRW^®^ Stem on Intracellular ROS Levels in Human Umbilical Cord Mesenchymal Stem Cells. The letters on the graph represent the statistical significance of differences; results sharing the same letter have a difference of *P* > 0.05 and results with different letters have a difference of *P* < 0.05.

In the control group, intracellular ROS levels were nearly undetectable (Fig. 6). Treatment with 300 μM H_2_O_2_ significantly increased ROS levels. The 5 μg/mL mitoquinol mesylate group showed no significant difference compared with the positive control group (*P* > 0.05). However, cells treated with 1 μg/mL mitoquinol mesylate, and 10 and 5 μg/mL SRW^®^ Stem exhibited significantly lower ROS levels than the positive control (*P* < 0.001), with 10 μg/mL SRW^®^ Stem showing the most pronounced effect, while 1 μg/mL mitoquinol mesylate and 5 μg/mL SRW^®^ Stem had comparable effects. These findings suggested that treatment with 1 μg/mL mitoquinol mesylate and 10 and 5 μg/mL SRW^®^ Stem may help hUC-MSCs resist H_2_O_2_-induced oxidative stress and maintain normal redox homeostasis.

**Fig. 7:**
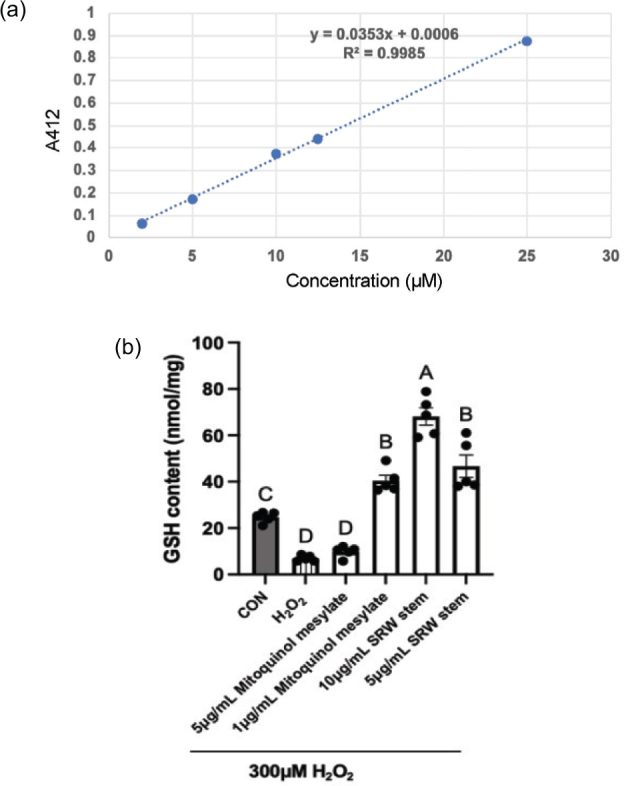
Effects of mitoquinol mesylate and SRW^®^ Stem on Total Glutathione Content in Human Umbilical Cord Mesenchymal Stem Cells. The letters on the graph represent the statistical significance of differences; results sharing the same letter have a difference of *P* > 0.05, and results with different letters have a difference of *P* < 0.05.

As shown in Fig. 7, the standard curve for total GSH exhibited an R^2^ value greater than 0.99, indicating a good fit for quantifying glutathione content. Based on this standard curve, GSH levels were calculated for each group. Treatment with 300 μM H_2_O_2_ significantly decreased intracellular GSH content (*P* < 0.01), indicating that cells exposed to oxidative stress lose redox homeostasis and are unable to maintain equilibrium through endogenous GSH antioxidant pathways. The GSH level in the 5 μg/mL mitoquinol mesylate group was similar to that in the 300 μM H_2_O_2_ group and significantly lower than that in the untreated control (*P* < 0.01), suggesting that 5 μg/mL mitoquinol mesylate does not promote GSH synthesis to counteract oxidative stress. In contrast, treatment with 1 μg/mL mitoquinol mesylate, and 10 and 5 μg/mL SRW^®^ Stem significantly increased intracellular glutathione levels compared to the 5 μg/mL mitoquinol mesylate group (*P* < 0.01), with the 10 μg/mL SRW^®^ Stem group showing the highest levels (68 nmol/mg). The effects of 1 μg/mL mitoquinol mesylate and 5 μg/mL SRW^®^ Stem were comparable (*P* = 0.60), which was consistent with the intracellular ROS levels.

### Effects on local inflammation

To investigate the anti-inflammatory effects of mitoquinol mesylate and SRW^®^ Stem, the levels of IL-1β and IL-10 in the cell culture supernatants of the hUC-MSCs under oxidative stress were measured.

**Fig. 8:**
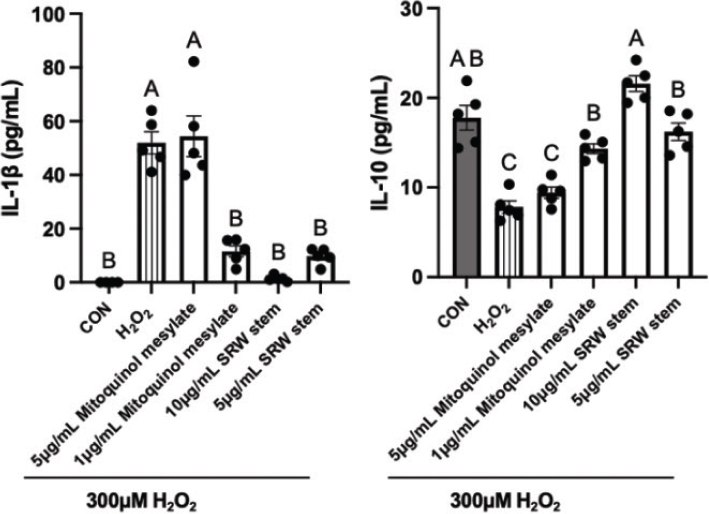
Effects of mitoquinol mesylate and SRW^®^ Stem on Inflammatory Cytokine Secretion by Human Umbilical Cord Mesenchymal Stem Cells. Graph (a and b) represent the effects on IL-1β and IL-10 concentrations, respectively. The letters on the graphs represent the statistical significance of differences, results sharing the same letter having a difference of *P* > 0.05 and results with different letters a difference of *P* < 0.05.

Under untreated conditions, the hUC-MSCs secreted negligible amounts of the pro-inflammatory cytokine IL-1β (Fig. 8). Stimulation with 300 μM H_2_O_2_ significantly increased IL-1β levels in the culture medium. Treatment with 5 μg/mL mitoquinol mesylate did not mitigate the H_2_O_2_-induced increase in IL-1β, with levels comparable to the 300 μM H_2_O_2_ group. In contrast, pretreatment with 1 μg/mL mitoquinol mesylate, and 10 and 5 μg/mL SRW^®^ Stem significantly reduced IL-1β secretion in response to H_2_O_2_ stimulation (*P* < 0.001), with IL-1β levels showing no significant difference from the control group (*P* > 0.05); notably, 10 μg/mL SRW^®^ Stem exhibited the most pronounced effect (1.42 pg/mL).

Concurrently, exposure to 300 μM H_2_O_2_ markedly decreased the secretion of the anti-inflammatory cytokine IL-10 by the hUC-MSCs. Treatment with 5 μg/mL mitoquinol mesylate did not alleviate this reduction. However, pretreatment with 1 μg/mL mitoquinol mesylate and 10 and 5 μg/mL SRW^®^ Stem significantly ameliorated the decrease in IL-10 secretion under oxidative stress (*P* < 0.001), with 10 μg/mL SRW^®^ Stem restoring IL-10 levels to near baseline.

## Discussion

SRW^®^ Stem showed statistically significant effects on cell formation, migration, differentiation, as well as anti-inflammatory and antioxidant effects in the hUC-MSCs. Notably, SRW^®^ Stem increased the colony formation rates of hUC-MSCs by approximately 30% (10 μg/mL) and 14% (5 μg/mL), increased the cell migration rate by approximately 213% (10 μg/mL) and 59% (5 μg/mL), promoted adipogenic differentiation, reduced intracellular ROS production by 80% (10 μg/mL) and 30% (5 μg/mL), promoted GSH synthesis, suppressed the production of the pro-inflammatory cytokine IL-1β, and enhanced the secretion of the anti-inflammatory cytokine IL-10. In all the tests, the effect of SRW^®^ Stem at a concentration of 10 and 5 μg/mL was stronger than mitoquinol at a concentration of 5 and 1 μg/mL. To further contextualise these findings, it is important to interpret the observed effects of SRW^®^ Stem in the light of the known molecular mechanisms governing stem cell function, particularly oxidative stress regulation, inflammatory signalling, and cellular senescence.

Stem cell proliferation, migration, and differentiation are tightly regulated by intracellular redox balance and mitochondrial function. Excessive accumulation of reactive oxygen species (ROS) is a well-established driver of stem cell dysfunction, leading to impaired self-renewal, reduced differentiation potential, and induction of cellular senescence pathways (12, 55). In this study, SRW^®^ Stem markedly reduced intracellular ROS levels while simultaneously increasing glutathione (GSH) synthesis, suggesting enhanced endogenous antioxidant systems. This is particularly relevant, as GSH is a central regulator of stem cell redox homeostasis and has been shown to preserve stemness and prevent premature senescence under oxidative stress conditions.

The observed antioxidant effects are likely mediated by complementary mechanisms of the individual ingredients. Mitoquinol, a mitochondria-targeted antioxidant, directly reduces mitochondrial ROS production and preserves mitochondrial membrane potential (34, 39), which is essential for maintaining stem cell energy metabolism and function. In parallel, sea buckthorn-derived proanthocyanidins have been shown to activate antioxidant defence pathways, including Nrf2 signalling, thereby enhancing cellular resistance to oxidative damage (40, 41). Vitamin D3 may further contribute by reducing oxidative DNA damage and supporting genomic stability (43). The combined action of these ingredients may therefore result in a more robust and sustained antioxidant response than mitoquinol alone, which is consistent with the superior effects observed in this study.

In addition to oxidative stress, chronic low-grade inflammation is a key driver of stem cell ageing and functional decline. Pro-inflammatory cytokines such as IL-1β impair stem cell proliferation and differentiation, while anti-inflammatory cytokines such as IL-10 support tissue repair and regenerative processes. The ability of SRW^®^ Stem to suppress IL-1β and restore IL-10 production under oxidative stress conditions suggests a shift toward an anti-inflammatory microenvironment that is more conducive to stem cell function. This effect is supported by previous studies demonstrating that fucoidan and oleuropein possess immunomodulatory properties, including inhibition of NF-κB signalling and reduced production of pro-inflammatory cytokines (42, 45). By attenuating inflammatory signalling, SRW^®^ Stem may help preserve the regenerative capacity of stem cells and prevent inflammation-induced senescence.

Importantly, the improvements observed in proliferation and colony-forming capacity indicate a potential effect on stem cell self-renewal. These processes are highly sensitive to both oxidative stress and inflammatory signalling but are also directly influenced by pathways involved in cellular ageing, such as the p53/p21 and p16INK4a pathways. Chronic activation of these pathways leads to irreversible growth arrest and senescence (55). The reduction in oxidative stress and inflammation observed in this study suggests that SRW^®^ Stem may indirectly modulate these senescence-associated pathways, thereby preserving proliferative capacity. This interpretation is consistent with previous findings showing that antioxidant and anti-inflammatory interventions can delay stem cell senescence and improve regenerative potential.

The enhanced migratory capacity observed with SRW^®^ Stem is also biologically significant, as stem cell migration is critical for tissue repair and regeneration. Migration is an energy-dependent process that relies heavily on mitochondrial function and cytoskeletal dynamics. The superior effect of SRW^®^ Stem compared to mitoquinol alone suggests that, beyond mitochondrial support, additional pathways may be involved. For example, fucoidan has been reported to upregulate CXCR4 expression (48), a key receptor involved in stem cell homing and migration, while polyphenolic compounds such as oleuropein may influence cytoskeletal remodelling and cell motility.

The promotion of adipogenic differentiation observed in this study further supports the role of SRW^®^ Stem in maintaining stem cell functionality. Differentiation capacity is known to decline with age due to epigenetic alterations, mitochondrial dysfunction, and senescence-associated changes (12, 55). The observed increase in lipid droplet formation suggests that SRW^®^ Stem helps preserve or restore differentiation potential under stress conditions. This finding aligns with previous studies demonstrating that fucoidan and oleuropein can modulate lineage-specific differentiation pathways in mesenchymal stem cells (45, 47). Although adipogenic differentiation was assessed in this study, these findings may reflect a broader capacity of SRW^®^ Stem to support multipotency.

hUC-MSCs were selected in this study due to their primitive developmental origin and well-documented regenerative potential compared with adult tissue–derived MSCs. hUC-MSCs are considered more ‘youthful’, exhibiting higher proliferative capacity, greater clonogenicity, and longer telomere length, alongside lower baseline levels of oxidative stress and senescence-associated markers (12, 55). These characteristics are particularly relevant to the present findings, as they may partly explain the robust responses observed in proliferation, migration, and differentiation assays. In addition, hUC-MSCs display strong paracrine and immunomodulatory activity, including the ability to modulate cytokine secretion profiles, which is consistent with the observed reduction in IL-1β and restoration of IL-10 under oxidative stress. Importantly, their relatively preserved mitochondrial function and redox balance may also make them more responsive to antioxidant interventions, such as those investigated in this study.

This study found that SRW^®^ Stem can improve various aspects of stem cell health, including proliferation, migration, and differentiation capacities, and can also reduce oxidative stress and inflammation in the hUC-MSC environment. This supports the potential of SRW^®^ Stem as both an anti-ageing product and an adjunct intervention in stem cell therapies. This study employed a wide range of methods to evaluate various aspects of stem cell health, making it the first comprehensive assessment of the effect of SRW^®^ Stem on stem cell health. The use of mitoquinol as an active control allows us to compare the whole formulation to one of its key ingredients, which is widely used in anti-ageing products.

Despite these strengths, this study has several limitations. Apart from mitoquinol, which was separated from the rest of the product, the other ingredients were not tested separately; therefore, it cannot be concluded that each ingredient contributed to the effects observed in this study. It is unclear if there are any additive or synergistic interactions among the ingredients in the product. Finally, how SRW^®^ Stem compares with other substances known to have stem cell health-promoting effects is unknown due to the lack of positive controls beyond mitoquinol. Future studies should compare the effect of SRW^®^ Stem to positive controls such as fibroblast growth factor-2 for proliferative function (56), transforming growth factor-β3 for differentiation (57) and stromal cell-derived factor-1 (58).

## Conclusions

This study shows that SRW^®^ Stem improved various aspects of stem cell health *in vitro*. These effects supported the potential of SRW^®^ Stem as an anti-ageing product. It will be important to confirm this potential in animal and human studies. Studies conducted on short-lived species, such as zebrafish and fruit flies, could be useful, as they allow us to follow the entire lifespan of an animal (59). Longitudinal studies in rodents that include multi-omic and behavioural assessments at multiple time points could also be useful (60). Human studies using biomarkers of ageing, such as DNA methylation, NAD/NADH, ATP, and telomere length (61) should be conducted to confirm the anti-ageing potential in humans. Finally, the potential role of SRW^®^ Stem in stem cell-based therapies, such as haematopoietic stem cell transplantation for blood cancers and stem cell therapy for macular degeneration, Parkinson’s disease, heart failure, and spinal cord injury, should be explored in future pre-clinical and clinical studies (62).
